# Differential testicular toxicity of deoxynivalenol: insights from juvenile and adult mice

**DOI:** 10.1007/s12550-026-00685-7

**Published:** 2026-10-06

**Authors:** Ana Laura, Paulino Leite Gomes, Juliana Rubira Gerez, Marielen de Souza, Rafaela Pires Erthal, Dayane Priscila dos Santos, Camila Rodrigues Ferraz, Waldiceu Aparecido Verri Junior, Eduardo Micotti da Gloria, Wellerson Rodrigo Scarano, Glaura Scantamburlo Alves Fernandes, Ana Paula Frederico Rodrigues Loureiro Bracarense

**Affiliations:** 1 Laboratory of Animal Pathology, Department of Preventive Veterinary Medicine, Universidade Estadual de Londrina, Londrina, Paraná Brazil; 2 Laboratory of Toxicology and Metabolic Reproductive Disorders, Department of General Biology, Universidade Estadual de Londrina, Londrina, Paraná Brazil; 3 Laboratory of Pain, Inflammation, Neuropathy and Cancer, Department of Immunology, Parasitology and General Pathology, Universidade Estadual de Londrina, Londrina, Paraná Brazil; 4 Biological Science Department, Luizde Queiroz College of Agriculture, Universidade de São Paulo, Piracicaba, São Paulo, Brazil; 5 Department of Structural and Functional Biology, Universidade Estadual Paulista (Unesp), Botucatu, Brazil

**Keywords:** Trichothecenes, Puberty, Infertility, Testicle, Spermatozoon

## Abstract

Deoxynivalenol (DON) is a mycotoxin that contaminates approximately 60% of food worldwide and can impair reproduction. Although puberty represents a critical window of susceptibility to toxic insults, the effects of DON exposure during this period remain poorly understood. This study evaluated the effects of DON on testicular structure, oxidative status, inflammatory responses, and sperm parameters in prepubertal and adult mice. Sixty mice were assigned to either a control diet or a DON-contaminated diet (10 mg/kg feed) and evaluated under two experimental regimens defined by developmental stage and exposure duration: prepubertal mice exposed for 15 days and adult mice exposed for 28 days. Testicular morphology and morphometry, caspase-3 immunohistochemistry, oxidative stress biomarkers, inflammatory profile, daily sperm production, and sperm morphology were assessed. DON induced morphological and morphometric alterations in the testes under both experimental regimens. The treatment altered the seminiferous epithelial cycle, reducing the proportion of tubules in stages VII–VIII. Caspase-3 expression was not affected by the treatment. DON impaired antioxidant defenses in both experimental regimens, as evidenced by reduced FRAP values and ABTS radical scavenging activity. Increased superoxide production was observed exclusively in prepubertal mice. The treatment increased the number of macrophages and IL-1β levels, whereas adult mice exhibited elevated TNF-α concentrations. In adults, DON reduced daily sperm production and increased sperm abnormalities. These findings demonstrate that both prepubertal and adult mice are susceptible to DON-induced reproductive toxicity.

**Graphical abstract:**

**Figure:**
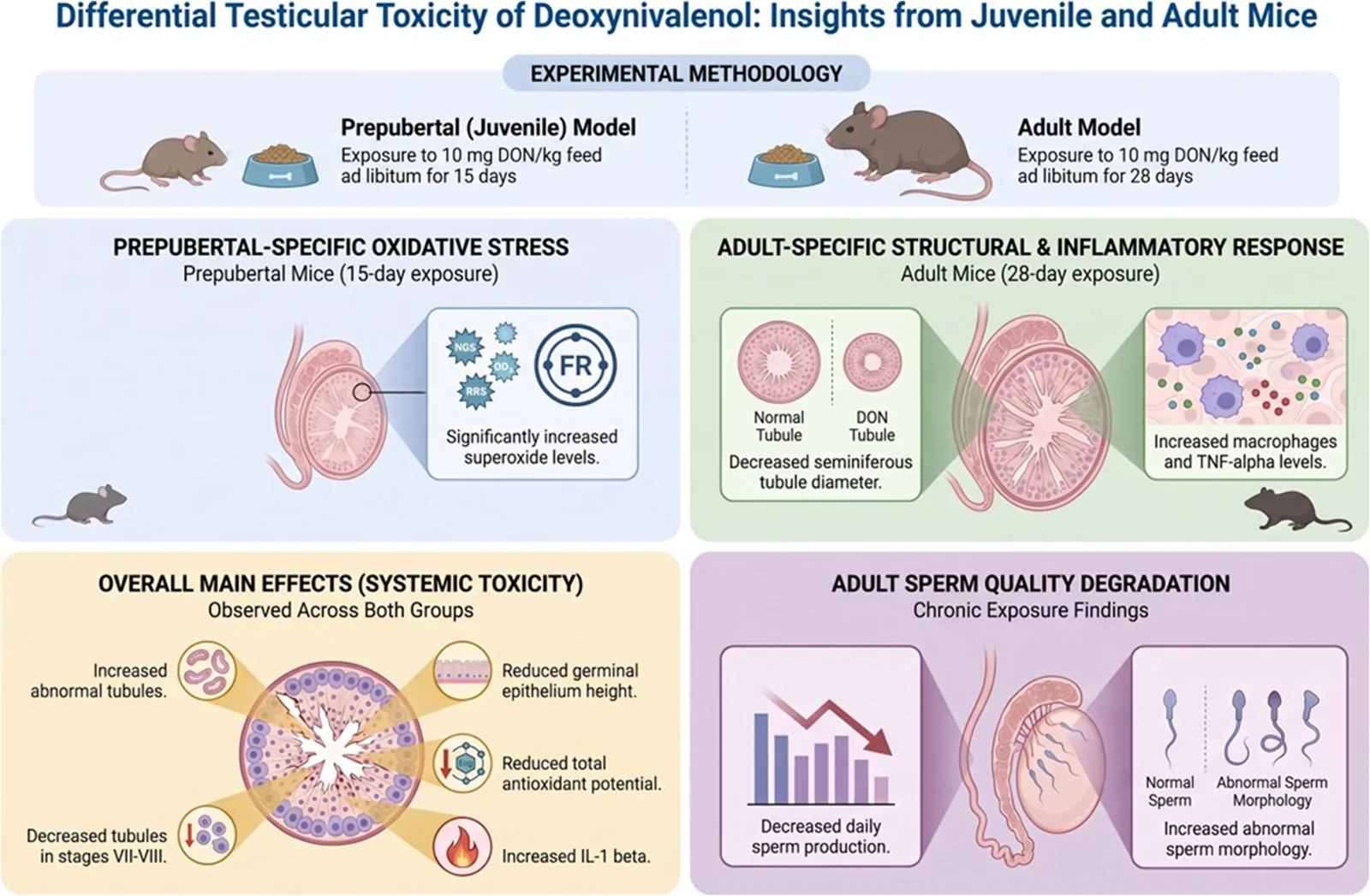

## Introduction

Male infertility is a prevalent condition, affecting approximately 55 million men of reproductive age (Zeng et al. 2025). This scenario parallels a marked global decline in sperm counts, estimated at 51.6% between 1973 and 2018 (Levine et al. 2023), and projections indicating a continued increase in male infertility rates up to 2040 (Liang et al. 2025). These trends have intensified the debate regarding the contribution of environmental contaminants to the progressive impairment of male fertility (Selvaraju et al. 2021). In this context, mycotoxins, toxic secondary metabolites produced by fungi that commonly contaminate grains and cereals, have been associated with disturbances in pubertal development and reduced reproductive performance in both animals and humans (Maranghi and Mantovani 2012; Okonofua et al. 2022).

In recent years, deoxynivalenol (DON), a widely occurring mycotoxin belonging to the type B trichothecenes, has received increasing attention due to its toxic potential on the male reproductive system (Dai et al. 2025; Gomes et al. 2025). Experimental studies using different animal models have demonstrated the toxic potential of DON on the adult male reproductive system. Exposure to DON at doses ranging from 1 to 12 mg/kg, for periods varying from 7 to 35 days, has been associated with multiple reproductive alterations in rodents. These effects include inhibition of steroidogenic enzymes (Yang et al. 2024), reduction in hormone levels (Sprando et al. 2005), decreased expression of tight junction proteins and disruption of the blood–testis barrier (BTB) (Miao et al. 2023). These effects are mainly mediated by oxidative stress (Yang et al. 2023, 2024), inflammatory response (Cao et al. 2020), activation of apoptotic pathways (Yang et al. 2019) and pyroptosis (Ruan et al. 2024). Such alterations culminate in a reduction in sperm quality and quantity, with consequent impairment of male fertility (Cao et al. 2020).

Oxidative stress is a key mechanism in DON-induced reproductive toxicity. DON exposure is associated with increased lipid peroxidation, evidenced by elevated malondialdehyde (MDA) levels (Yang et al. 2019; Li et al. 2023), higher testicular arachidonic acid content (Yang et al. 2023), and upregulation of lipid peroxidation-related genes (ACSL4, LPCAT3, PTGS2)(Yang et al. 2023) in mice exposed to 2.0-2.4 mg/Kg for approximately one month. This is accompanied by increased reactive oxygen species (ROS) and hydrogen peroxide production in both in vitro and in vivo models, including Leydig cells exposed to DON(Sun et al. 2022; Yang et al. 2024) and mice treated with 1–2.4 mg/kg DON for up to 28–30 days (Yang et al. 2019, 2023). Concomitantly, in mice exposed to 2.0-2.4 mg/Kg for approximately one month, antioxidant defense systems are suppressed, with reduced activity of SOD and CAT (Yang et al. 2019, 2023), iron homeostasis disrupted, Nrf2-Keap1 pathway altered and disruption of the Xc⁻/GPX4 axis, including decreased GPX4 expression, GSH content, and GSH/GSSG ratio (Yang et al. 2023).

Furthermore, DON induces an inflammatory response in the male reproductive system, characterized by the upregulation of pro-inflammatory cytokines and signaling pathways. Increased mRNA expression of TNF-α, IL-1α, and IL-6 has been reported in mice exposed to 2.4 mg/kg DON for 28 days by oral gavage (Cao et al. 2020), together with upregulation of inflammation-related genes such as Tnfrsf9, Rnf144b, Itga9, IL6st, and Relb in mice fed 12 mg/kg DON for 35 days (Li et al. 2023). In addition, DON promotes the release of inflammatory cytokines, including IL-1β and IL-18, as observed in mice exposed to 2.3 mg/kg for 21 days (Ruan et al. 2024), suggesting activation of inflammasome-related pathways.

Despite advances in understanding the reproductive effects of DON in adult males, a recent review conducted by our group identified a major gap related to the scarcity of studies evaluating the effects of this mycotoxin in young individuals, with only two studies currently available (Gomes et al. 2025). Gerez et al. (2021) examined the testes of prepubertal Wistar rats exposed to feed with 9.4 mg DON/kg feed for 28 days and reported a reduction in Sertoli and Leydig cell numbers, along with altered spermatogenic dynamics and increased cell proliferation. In addition, Yang et al. (2024) investigated progenitor Leydig cells in Sprague–Dawley rats exposed to 2 and 4 mg/kg DON and observed changes in serum hormone levels, with decreased testosterone and increased luteinizing hormone (LH), accompanied by oxidative stress, enhanced mitochondrial fission, and stimulation of Leydig progenitor cell proliferation associated with impaired maturation (Yang et al. 2024).

Elucidating the effects of DON on the male reproductive system during the prepubertal period is particularly relevant, as this developmental window is characterized by intense morphological and hormonal changes that confer increased vulnerability to toxic insults (Fisher and Eugster 2014). This concern is further reinforced by the high frequency of urinary DON detection in children and adolescents, with estimates indicating that 33–63% of children and 5–46% of adolescents exceed the provisional tolerable daily intake (TDI) for this mycotoxin (Papageorgiou et al. 2018). Although the doses associated with reproductive toxicity in experimental studies are substantially higher than the established TDI for DON (1 µg/kg body weight/day) (Papageorgiou et al. 2018), the frequent exceedance of this threshold in children and adolescents raises concerns regarding chronic exposure during critical developmental periods. Therefore, this study aimed to evaluate the toxic effects of DON on the testes and spermatozoa of prepubertal, and adult mice exposed to oral doses of 10 mg DON/kg feed for 15 and 28 days, respectively.

## Materials and methods

### Ethical statement

All experimental procedures were conducted in accordance with the guidelines for the ethical use of animals in research and were approved by the Ethics Committee on Animal Use (CEUA) of the State University of Londrina (UEL), under protocol number 27/2019, and were conducted in accordance with Brazilian regulations. In addition, the study complied with the European Directive 2010/63/EU and followed ARRIVE guidelines.

### Animals and experimental conditions

Male Swiss mice were sourced from the Central Animal Facility of the State University of Londrina (UEL, BR). These animals were maintained in micro isolating polycarbonate boxes measuring 41 × 34 × 16 cm, housing five animals per cage, equipped with stainless steel wire grids, feeders and a drinking trough. Bedding consisted of high-quality autoclaved pine shavings. Throughout the experiment, the animals received a specific balanced rodent diet (Nuvilab CR-1, Quimtia S/A) and water *ad libitum*. The experimental place had temperature controls (22 ± 2 °C), ventilation, photoperiod (12 h of light for 12 h of dark) and relative humidity (between 50 and 55%).

### Preparation and analysis of experimental diets

The Laboratory of Mycology, Luiz de Queiroz College of Agriculture, University of São Paulo (Piracicaba, SP, Brazil) produced a crude DON extract that was obtained through solid-state fermentation using known DON-producing strains of *Fusarium graminearum*. Fungal inocula were prepared by culturing the strains on potato dextrose agar for 7 days at 25 °C. The cultures were used to inoculate autoclaved, moistened rice. Fermentation was carried out in 500 mL Erlenmeyer flasks containing 100 g moistened rice, each inoculated with a 2-mL of a spore suspension (1 × 105 spores/mL). The flasks were maintained under static conditions at 25 ◦C for 28 days. After the fermentation period, the rice substrate was dried, ground, and analyzed to determine the mycotoxin concentration.

Quantification of DON was performed using HPLC, revealing a concentration of 770.2 mg/kg. A blend (standard diet + DON) was prepared at State University of Londrina facilities using a commercial feed mixer (Gerez et al. 2022). It should be noted that the use of the crude extract does not rule out the presence of other toxins; however, DON was the predominant compound detected. Moreover, studies using crude extracts tend to better reflect real-world contamination scenarios.

The contaminated diet was adjusted according to the DON concentration present in the crude extract to obtain a final concentration of 10 mg DON/kg of feed. At the beginning and end of the experimental period, diet samples were sent to the LAMIC Laboratory (Santa Maria – RS, Brazil) for mycotoxins analysis via HPLC/MS-MS. The control diet contained DON levels below the established limit (50 µg/kg with a stated accuracy of 101%) (Mallmann et al. 2020), based on the European Legislation for processed cereal-based foods and baby foods for infants and young children (Commission Regulation (EC) No 1881/2006). In addition, other mycotoxins were analyzed, including aflatoxin (AFB1, AFB2, AFG1, AFG2), zearalenone (ZEA) and fumonisins (FB1, FB2), which remained below the detection limits of 1 µg/kg (AFB1, AFB2, AFG1, AFG2), 20 µg/kg (ZEA) and 125 µg/kg (FB1, FB2), respectively.

### Experimental design and biological sample collection

Thirty prepubertal male mice, aged 21 postnatal days, and thirty adult male mice, aged 65 postnatal days, were included in the experiment. These ages were selected based on established developmental parameters for mice, according to which the peripubertal period ends at approximately postnatal day 35 (Picut et al. 2018), whereas full adulthood is reached after postnatal day 70 (Drumond et al. 2011). Within each developmental group, animals were randomly assigned to receive either a control diet or a diet containing 10 mg DON/kg feed. Each treatment group comprised 15 prepubertal and 15 adult mice.

Animals assigned to the same treatment were housed in pairs, with both animals within each cage receiving the same experimental diet. Therefore, the cage represented the experimental unit for dietary exposure. Animals had *ad libitum* access to feed and water throughout the experimental period, which lasted 15 days for prepubertal mice and 28 days for adult mice. Because age at the beginning of exposure and exposure duration differed simultaneously, these experimental conditions were defined as the prepubertal/15-day and adult/28-day experimental regimens.

The selected DON concentration corresponds to concentrations occasionally detected in food products, animal feed, and feed ingredients (5–25 mg/kg feed) and is considered sublethal (Grenier and Applegate 2013). This exposure level has previously been employed in experimental studies and shown to induce reproductive toxicity without evident systemic toxicity (Gerez et al. 2021; Lemos et al. 2024; Kono et al. 2026).

No mortality or clinical signs of toxicity were observed during the experimental period. At the end of each exposure period, animals were premedicated with acepromazine (2.5 mg/kg, intramuscularly) and deeply anesthetized with thiopental (60 mg/kg, intraperitoneally). The two animals housed within each cage were then allocated to different sets of analyses.

One animal from each cage was euthanized by decapitation and assigned to histological and reproductive evaluations. The right testis was collected and fixed in Methacarn solution, consisting of 60% methanol, 30% chloroform, and 10% glacial acetic acid, for histopathological, morphometric, and immunohistochemical analyses. In adult mice, the left testis was collected for the determination of daily sperm production. Spermatozoa used for morphological evaluation were recovered from the *vas deferens* by internal lavage with 1.0 mL of 10% formalin–saline solution.

The second animal from each cage was assigned to biochemical analyses. While under deep anesthesia, blood was collected by cardiac puncture, followed by transcardial perfusion with saline solution to remove residual blood from the testicular tissue and minimize blood-derived interference in the biochemical assays. Following perfusion, the right testis was collected for assessment of oxidative status using the FRAP, ABTS, NBT, and TBARS assays. The left testis was collected for evaluation of the inflammatory profile by measuring NAG activity and the concentrations of IL-1β and TNF-α. Both testes were stored at − 80 °C until the respective biochemical analyses were performed.

Thus, histological and reproductive evaluations and biochemical measurements were performed using different animals from the same experimental cage. Only one animal from each cage contributed to each set of analyses, thereby preventing the two animals sharing the same cage from being treated as independent experimental replicates for the same outcome.

### Zootechnical parameters

Final body weight was measured at the cage level at the end of each experimental period. The combined weight of the two animals housed in each cage was divided by two to obtain the cage-mean body weight per animal, expressed as g/animal. The cage-derived means were used as the independent observations to calculate the group means and standard deviations and to perform the statistical analysis. Thus, the cage, rather than the individual animal, was considered the experimental unit for final body weight.

### Histopathological and morphometric analysis

The right testes were processed according to routine histological techniques and stained with hematoxylin and eosin (Wick 2019). Histological analyses were performed using an optical microscope (O400S, Opticam Microscopy Technology Global Company, United States), and, when necessary, analyzed in an image processing system (OPTHD software, Opticam Microscopy Technology Global Company, United States). These slides were used to evaluate the seminiferous tubules morphology, dynamics of spermatogenesis; to measure the diameters of the seminiferous tubules and the height of the spermatogenic epithelium; and to count Sertoli and Leydig cells.

Histological analysis was performed in 100 randomly selected testicular cross-sections per animal. The seminiferous tubules were classified into normal or abnormal. Abnormal tubules had immature germ cells in the lumen; acidophilic cells; cytoplasmic vacuolization; cell degeneration (seminiferous epithelium disruption with absence of germ cells and loss of normal tissue architecture) and the presence of multinucleated cells (Erthal et al. 2020).

The dynamics of spermatogenesis was performed by classifying one hundred random tubular sections per animal into four categories: stages I-VI (two generations of spermatids - rounded and elongated), VII-VIII (presence of spermatozoa in the lumen), IX-XI (one generation of spermatid - only rounded or only elongated) and XII (presence of secondary spermatocyte) of the seminiferous epithelium cycle, according to Leblond and Clermont (1952).

The diameter of the seminiferous tubules and the height of the germinal epithelium were assessed in ten random seminiferous tubules at stage IX of spermatogenesis dynamics for each animal. Four diameter and height measurements were obtained for each tubule; the averages were calculated and used in the statistical analysis for comparison between groups (Erthal et al. 2020).

The number of Sertoli cell nuclei was determined in 20 cross sections of seminiferous tubules at stages VII-VIII of spermatogenesis, in each testis. The number of Leydig cell nuclei was counted in 10 random fields, at 400x magnification, of the interstitial tissue in each testicular section per animal (Erthal et al. 2020).

### Immunohistochemical analysis

The expression of caspase-3 (Cleaved caspase-3 antibody [Asp175], 1:100 dilution, Cell Signaling Technology, Massachusetts, USA), an effector endoprotease of apoptosis, was evaluated by immunohistochemical analysis in the testicular tissue. Silanized slides containing 3-micrometer sections were subjected to deparaffinization and rehydration in a series of alcohols. Antigen retrieval was performed in a pressure cooker for 45 min using citrate buffer pH 6.0. Endogenous peroxidase was blocked by incubating the slides with a 97% methanol and 3% hydrogen peroxide solution, and a blocking solution was used for non-specific binding. The sections were incubated for 22 h in a humid chamber at 4 °C with the primary antibody caspase-3 (Cleaved caspase-3 antibody [Asp175], 1:100 dilution, Cell Signaling Technology, Massachusetts, USA). The slides were then incubated with the polymer-linked secondary antibody (Polydector) (Super Picture™ Polymer Detection Kit; Invitrogen Corporation, Camarillo, CA, USA) for two hours. The final reaction was performed with 3,3’-diaminobenzidine tetrahydrochloride (DAB) and stained with hematoxylin. Spleen sections were used to control the reaction.

The analysis was performed based on the frequency of immunostaining in different testicular cell populations. For this purpose, a score was assigned based on the percentage of immunoreactive cells, according to the following classification: 0 (absence of staining), 1 (staining in 1–25% of cells), 2 (staining in 26–50%), 3 (staining in 51–75%), and 4 (staining in 76–100%). Scores were determined individually for the following testicular cells: spermatogonia, spermatids, Sertoli cells, and Leydig cells.

### Oxidative stress response assay

Initially, samples were homogenized in ice-cold KCl buffer solution (1.15%), and the resulting supernatant was used for subsequent analyses.

The antioxidant potential was determined by ferric ion reduction assay (FRAP) and free radical scavenging capacity assay (ABTS) as described by Katalinic et al. (2005).In the FRAP assay, the supernatant was incubated with the FRAP reagent (Sigma Chemical Co., St. Louis, MO, USA) at 37 °C for 30 min, and the absorbance was measured at 595 nm. For the ABTS assay, the supernatant was mixed with a solution containing the reagent 2,2-azino-bis-(3-ethylbenzothiazoline-6-sulfonic acid) (Sigma Chemical Co., St. Louis, MO, USA) and, after incubation for 6 min at 25 °C, the absorbance was determined at 730 nm. The results of both assays were compared to a Trolox standard curve (0.02–20 nmol) and expressed as nmol of Trolox equivalent per mg of protein.

Superoxide anion production was quantified by nitro blue tetrazolium (NBT) reduction, as described by Fattori et al. (2017). The supernatant was incubated with NBT (Amresco, Solon, OH, USA) at 37 °C in a water bath for 5 min. Next, the supernatant was removed, and the formazan formed was solubilized by the addition of 2 M KOH (Sigma Chemical Co., St. Louis, MO, USA) and dimethyl sulfoxide (DMSO; Sigma Chemical Co., St. Louis, MO, USA). NBT reduction was determined by absorbance at 600 nm, and the results were expressed as optical density per milligram of protein.

Lipid peroxidation was measured by determining the levels of thiobarbituric acid reactive substances (TBARS), according to the method adapted from Guedes et al. (2006). Trichloroacetic acid (10%) was added to the homogenate, followed by centrifugation (1000 × g, 3 min, 4 °C). After separation, the protein-free supernatant was incubated with thiobarbituric acid (0.67%) in a water bath at 100 °C for 15 min. MDA levels, an intermediate product of lipid peroxidation, were determined by the difference between absorbances at 535 and 572 nm, with results expressed as nmol of MDA per mg of protein.

All spectrophotometric analyses were performed using a microplate reader (Multiskan GO Microplate Spectrophotometer, Thermo Scientific, Vantaa, Finland).

### Inflammatory profile

The inflammatory profile was evaluated in the testes of prepubertal and adult mice by quantifying macrophages, by the activity of N-acetyl-β-D-glucosaminidase (NAG), and by determining the levels of inflammatory cytokines interleukin 1 beta (IL-1β) and tumor necrosis factor alpha (TNF-α).

NAG enzymatic activity was determined as described by Erthal et al. (2020). Samples were homogenized and centrifuged (16,100 × g, 2 min, 4 °C), and aliquots of the supernatant (20 µL) were distributed into 96-well plates and diluted in K₂HPO₄ buffer (80 µL, 50 mM, pH 6.0). The reaction was initiated by adding K₂HPO₄ buffer (100 µL, 50 mM, pH 6.0) containing the substrate 4-nitrophenyl N-acetyl-β-D-glucosaminide (2.24 mM). After incubation at 37 °C for 10 min, the reaction was stopped by adding glycine buffer (100 µL, 0.2 M, pH 10.6). Enzyme activity was determined by spectrophotometry at 400 nm using a microplate reader (Multiskan GO, Thermo Scientific, Vantaa, Finland). The results were expressed as the number of macrophages per mg of tissue, based on a macrophage standard curve (196–400,000 cells).

For cytokine analysis, testes were homogenized in sterile saline solution (0.9% NaCl) using a Polytron^®^ homogenizer (PT3100, Kinematica AG, Lucerne, Switzerland) and centrifuged at 3600 rpm for 15 min. The resulting supernatant was analyzed by enzyme-linked immunosorbent assay (ELISA) using commercial kits (R&D Systems, Minneapolis, MN, USA), according to the manufacturer’s instructions, for the quantification of IL-1β (sensitivity of 8 pg/mL) and TNF-α (sensitivity of 4 pg/mL). Cytokine levels were expressed in pg per 100 mg of tissue.

### Daily sperm production and sperm morphology

In adult animals, mature spermatid counts and analysis of the daily sperm production (DSP) were performed (Siervo et al. 2023). The testes were homogenized, and the contents transferred to a Neubauer chamber, where spermatids resistant to homogenization were counted. DSP was calculated by dividing the number of mature spermatids present in the testis by 4.84 (the number corresponding to the number of days the mature spermatids were in the seminiferous epithelium).

The quality of the sperm was also analyzed. The sperm was obtained by washing the *vas deferens* and posteriorly preparing smears with this solution. The morphology of one hundred random spermatozoon from each animal was analyzed. Spermatozoa were classified as: normal morphology, head abnormalities (without characteristic curvature or isolated shape, without attached tail) and tail abnormalities (broken, spirally coiled and isolated, without attached head) (Fernandes et al. 2007).

### Statistical analysis

Data are presented as mean ± standard deviation (SD). The residual normality of residues was assessed using the Shapiro–Wilk test, and homogeneity of variances was evaluated using Bartlett’s test. When these assumptions were not met, data were logarithmically transformed and model assumptions were reassessed. After residual normality and homoscedasticity had been achieved, the data were analyzed using two-way analysis of variance. For outcomes evaluated in both experimental regimens, statistical analyses followed a 2 × 2 factorial design, with treatment (Control or DON), experimental regimen (prepubertal/15-day or adult/28-day), and the treatment-by-regimen interaction included as fixed effects. When a significant treatment-by-regimen interaction was detected (*p* ≤ 0.05), Šídák-adjusted simple-effects comparisons were performed to compare the control and DON groups within each experimental regimen (*p* ≤ 0.05). In the absence of a significant interaction, the main effects of treatment and experimental regimen were interpreted (*p* ≤ 0.05).

Seminiferous epithelium dynamics was analyzed separately because the numbers of tubules classified into stages I–VI, VII–VIII, IX–XI, and XII represented mutually exclusive counts that summed to 100 for each animal. Therefore, the four stage categories were treated as a single composition, with the animal considered the experimental unit. Stage proportions were transformed into isometric log-ratio coordinates, and the overall effects of treatment, experimental regimen, and their interaction were assessed using multivariate permutation tests based on the Freedman–Lane procedure. Complementary permutation-based factorial analyses were subsequently performed for each stage category to identify which categories contributed to the overall differences. The resulting stage-specific *p*-values were adjusted for the four comparisons using the Holm procedure. Holm-adjusted *p* ≤ 0.05 was considered statistically significant.

For parameters evaluated exclusively in adult animals (DSP and sperm morphology), Student’s t-test was applied when parametric assumptions were met, with results expressed as mean ± SEM. Otherwise, the Mann-Whitney test was used, with results expressed as median [Q1–Q3]. Statistical significance was set at *p* < 0.05.

## Results

### Zootechnical parameters

Regarding the assessment of final body weight, significant interactions between regimen and treatment were detected (*p* = 0.001). Comparisons adjusted using the Šídák method revealed a significant reduction in weight among animals treated with DON under the peripubertal regimen (Table 1).

**Table 1:** Final body weight of prepubertal and adult mice fed a control or DON-contaminated diet for 15 and 28 days, respectively

Experimental regimen	Prepubertal Control	Prepubertal DON	Adult Control	Adult DON	Mean Control	Mean DON	Treatment effect *p*-value	Regimen effect *p*-value	Treatment × regimen *p*-value
Final body weight (g/animal)	33.10 ± 2.22	24.50 ± 2.12*	43.17 ± 2.93	41.14 ± 1.84	38.13 ± 5.80	32.82 ± 8.84*	< 0.0001	< 0.0001	0.001

Values are expressed as mean ± standard deviation. Final body weight is presented as the cage-mean body weight per animal (g/animal, with the cage considered the experimental unit. *p*-values for the effects of treatment, experimental regimen, and the treatment-by-regimen interaction were obtained using two-way ANOVA. Because a significant interaction was detected, the control and DON groups were compared within each experimental regimen using Šídák’s multiple-comparisons test. The asterisk (*) indicates a significant difference compared with the corresponding prepubertal Control group (*p* < 0.0001)

### Histopathological and morphometric analysis

**Fig. 1:**
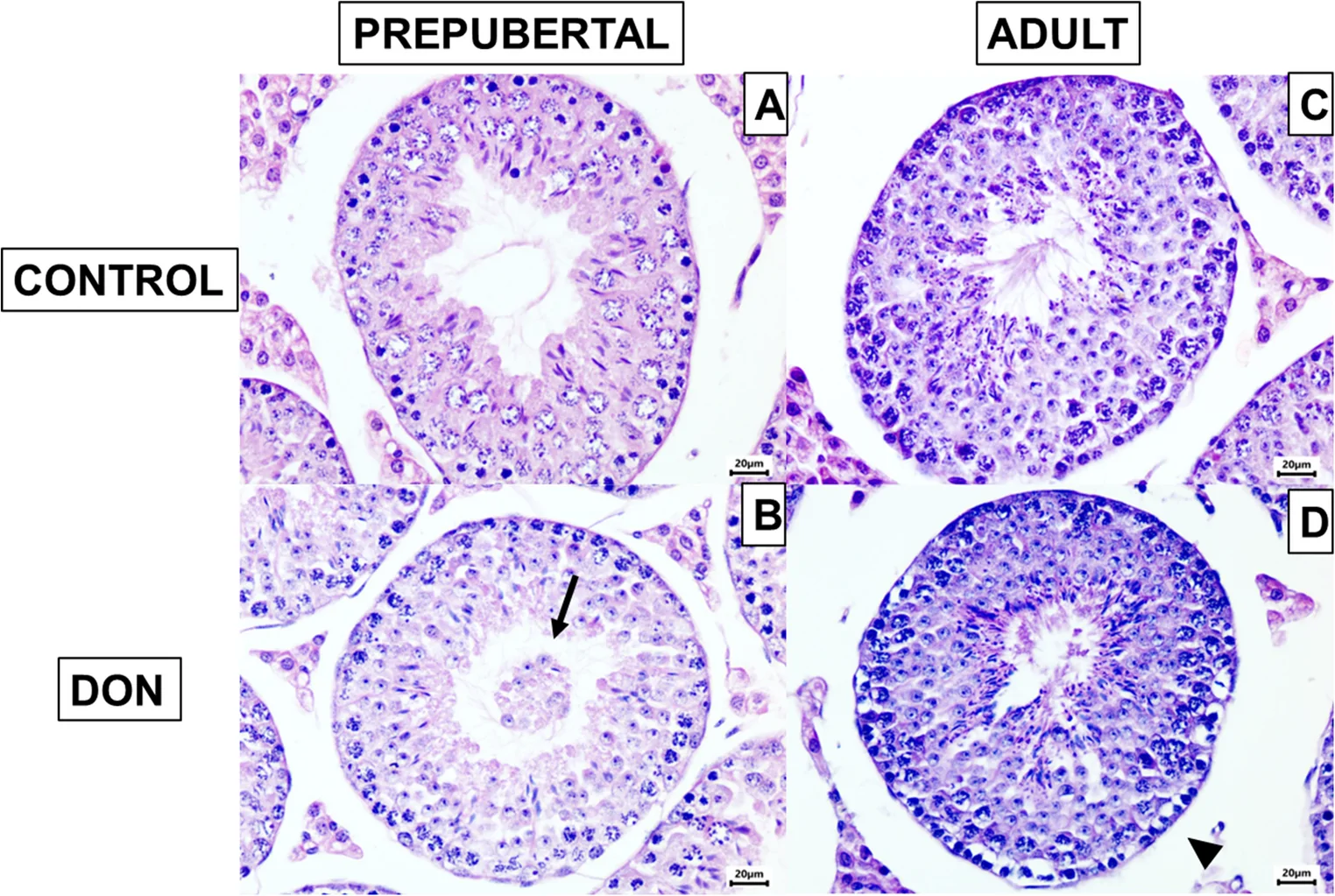
Histological changes in the testes of mice exposed to a control or DON-contaminated diet. A and B – Prepubertal mice. **A** Control group. Well-differentiated seminiferous tubules in stages I-VI. **B** DON group. Seminiferous tubules in stages I-VI with rounded cells in the lumen (arrow). **C** and **D** – Adult mice. **D** Control group. Well-differentiated seminiferous tubules in stages VII-VIII. D) DON group. Seminiferous tubules in stages VII-VIII with vacuolation (arrowhead) of the germinal epithelium. Hematoxylin-eosin staining. Scale bar: 20 μm

Considering the DON toxicity on male reproduction, we performed the morphological analysis of the testes. Exposure of the testes to DON significantly increased the number of abnormal seminiferous tubules when both experimental regimens were considered together (*p* = 0.0027), with no interaction between regimen and treatment (*p* = 0.48) (Table 2). The main alterations observed were the presence of cells in the tubular lumen, cytoplasmic vacuolization, and acidophilic cells (Fig. 1). We observed that DON significantly reduced the proportion of seminiferous tubules in stages VII–VIII when considering both experimental regimens together (*p* = 0.0029), with no interaction between regimen and treatment (*p* = 0.3750) (Table 3).

**Table 2:** Histopathological and morphometric analysis in testes of prepubertal and adult mice exposed to a diet exposed to a control or DON-contaminated diet for 15 days and 28 days, respectively

Analysis	Prepubertal Control	Prepubertal DON	Adult Control	Adult DON	Mean Control	Mean DON	Treatment effect *p*-value	Regimen effect *p*-value	Treatment × regimen *p*-value
Lesion score	1.50 ± 1.05	4.80 ± 1.79	4.80 ± 2.05	9.80 ± 4.71	3.00 ± 2.28	7.30 ± 4.27*	0.0027	0.0027	0.48
Seminiferous tubule diameter (µm)	121.61 ± 5.44	121.80 ± 5.71	137.28 ± 5.97	121.30 ± 11.54*	128.84 ± 9.80	121.55 ± 8.59*	0.0081	0.011	0.0069
Germinal epithelium height (µm)	40.42 ± 3.36	37.91 ± 4.16	45.98 ± 3.73	42.31 ± 3.67	42.95 ± 4.43	40.11 ± 4.37*	0.032	0.0013	0.67
Number of Sertoli cells per seminiferous tubule	11.02 ± 1.31	12.05 ± 1.51	10.12 ± 2.67	10.64 ± 0.88	10.61 ± 1.98	11.41 ± 1.41	0.21	0.07	0.67
Number of Leydig cells (10⁻³ mm²)	20.88 ± 3.19	19.56 ± 2.56	16.63 ± 2.41	14.56 ± 2.47	18.95 ± 3.51	17.29 ± 3.54	0.08	< 0.0001	0.98

Values are expressed as mean ± standard deviation. “Mean Control” and “Mean DON” represent the respective means across the prepubertal and adult experimental regimens. *p*-values for the effects of treatment, experimental regimen, and the treatment-by-regimen interaction were obtained using two-way ANOVA. When a significant interaction was detected, the control and DON groups were compared within each experimental regimen using Šídák’s multiple-comparisons test.*p < 0.05; ***p < 0.0001

**Table 3:** Dynamics of spermatogenesis, according to Leblond and Clermont (1952), in prepubertal and adult mice exposed to a control or DON-contaminated diet for 15 and 28 days, respectively

Seminiferous tubule stages	Prepubertal Control	Prepubertal DON	Adult Control	Adult DON	Mean Control	Mean DON	Treatment effect Adjusted *p*	Regimen effect Adjusted *p*	Treatment × regimen Adjusted *p*
I–VI	39.50 ± 6.98	46.00 ± 8.12	36.60 ± 4.16	39.60 ± 6.84	38.05	42.80	0.2524	0.4012	1.0000
VII–VIII	31.50 ± 5.32	18.40 ± 5.32	34.40 ± 5.41	28.80 ± 2.59	32.95	23.60*	0.0029	0.0259	0.3750
IX–XI	23.50 ± 4.32	30.00 ± 5.39	23.80 ± 3.19	26.40 ± 5.03	23.65	28.20	0.1103	0.8285	1.0000
XII	5.50 ± 1.52	5.60 ± 1.95	5.20 ± 1.48	5.20 ± 1.30	5.35	5.40	0.9416	0.8285	1.0000

Values are expressed as mean ± standard deviation. One hundred seminiferous tubule cross-sections were classified into four mutually exclusive stage groups for each animal. Following the global compositional analysis, complementary permutation-based factorial analyses were performed for each stage category. The reported *p*-values were adjusted for the four stage-specific analyses using the Holm procedure. “Mean of Control” and “Mean of DON” represent the respective means across the prepubertal and adult experimental regimens, with equal weight assigned to each regimen. The asterisk (*) indicates a significant difference between the mean of DON-treated animals and the mean of control animals for stages VII–VIII (Holm-adjusted *p* = 0.0029)

Seminiferous tubule diameter was significantly affected by treatment (*p* = 0.0081) and experimental regimen (*p* = 0.011), with a significant interaction between treatment and regimen (*p* = 0.0069). Consequently, a significant reduction in seminiferous tubule diameter was observed in adult animals. On the other hand, germinal epithelium height was reduced when both experimental regimens were considered together (*p* = 0.032), with no interaction between regimen and treatment (*p* = 0.67) (Table 2).

Sertoli and Leydig cell count showed no significant changes due to treatment (*p* = 0.21 and *p* = 0.08, respectively) and no significant interaction between treatment and regimen (*p* = 0.67 and *p* = 0.98, respectively) (Table 2).

### Immunohistochemical analysis

**Fig. 2:**
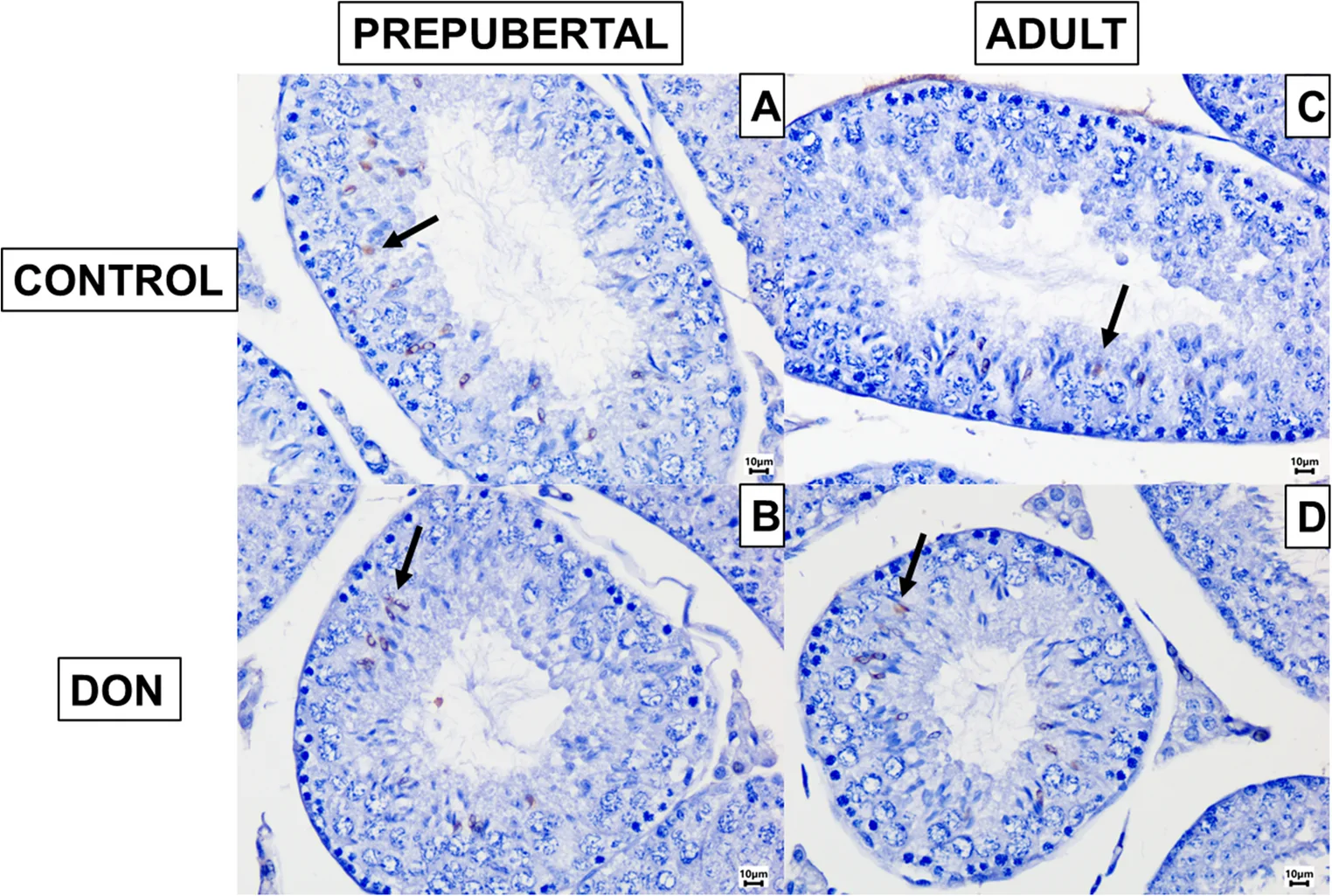
Immunohistochemical analysis of caspase-3 in the testes of mice exposed to a control or DON-contaminated diet. **A** Prepubertal mice; control group. **B** Prepubertal mice; DON group. **C** Adult mice; control group. **D** Adult mice; DON group. Figures A, B, D, E – Seminiferous tubules showing caspase-3 immunostaining in spermatids (arrows), corresponding to score 1. Scale bar: 10 μm

In the testes of prepubertal and adult mice, caspase-3 immunostaining was observed in spermatids (Fig. 2) and Leydig cells, with a frequency corresponding to score 1 (1–25% staining). However, no statistically significant differences in immunoreactivity were detected (*p* = 1).

### Oxidative stress response assay

The toxic effects of DON on various animal species occur by affecting different biological systems, such as the induction of oxidative stress (Ndlovu et al. 2021). We observed that DON significantly reduced FRAP (*p* = 0.0002) and ABTS (*p* = 0.0013) values when both experimental regimens were considered jointly, without regimen x treatment interaction (*p* = 0.22 and *p* = 0.23, respectively). For the assessment of superoxide anion production, significant regimen × treatment interactions were detected for NBT (*p* = 0.0086). The Šídák-adjusted comparisons for NBT showed a significant increase in superoxide anion levels in DON-treated animals only in the peripubertal regimen. In contrast, TBARS levels showed no significant effect of treatment (*p* = 0.21) or treatment × regimen interaction (*p* = 0.64) (Table 4).

**Table 4:** Oxidative stress in testes of prepubertal and adult mice exposed to a diet exposed to a control or DON-contaminated diet for 15 days and 28 days, respectively

Analysis	Prepubertal Control	Prepubertal DON	Adult Control	Adult DON	Mean Control	Mean DON	Treatment effect *p*-value	Regimen effect *p*-value	Treatment × regimen *p*-value
FRAP (nmol Trolox Eq./mg protein)	22.02 ± 3.09	16.80 ± 4.09	23.34 ± 2.93	14.16 ± 1.96	22.85	15.57***	0.0002	0.67	0.22
ABTS (nmol Trolox Eq./mg protein)	95.98 ± 10.11	66.12 ± 8.84	23.42 ± 15.00	8.60 ± 5.83	59.70	37.40*	0.0013	< 0.0001	0.23
NBT (OD/mg protein)	1.24 ± 0.35	2.38 ± 0.93*	1.28 ± 0.63	0.98 ± 0.44	1.26	1.62*	0.011	0.012	0.0086
TBARS (nmol MDA/mg protein)	2.08 ± 0.54	1.83 ± 0.41	2.20 ± 1.19	1.66 ± 0.43	2.14	1.75	0.21	0.94	0.64

Values are expressed as mean ± standard deviation. “Mean Control” and “Mean DON” represent the respective means across the prepubertal and adult experimental regimens. *p*-values for the effects of treatment, experimental regimen, and the treatment-by-regimen interaction were obtained using two-way ANOVA. When a significant interaction was detected, the control and DON groups were compared within each experimental regimen using Šídák’s multiple-comparisons test.*p < 0.05; ***p < 0.0001. FRAP - Iron ion reducing power; ABTS − 2,2’-azino-bis (3-ethylbenzothiazoline-6-sulfonate); NBT – Nitroblue tetrazolium reduction assay; TBARS: Thiobarbituric acid reactive substances level

### Inflammatory profile

In vitro and in vivo studies have demonstrated that the inflammatory capacity of DON induces the release of cytokines (Melagoda et al. 2019; Cao et al. 2020). The results showed significant interactions between regimen and treatment for macrophage quantification (NAG) (*p* = 0.017) and TNF-α levels (*p* = 0.04). When these comparisons are adjusted using the Šídák method, NAG and TNF-α indicated a significant increase only in adult animals treated with DON. Furthermore, it was observed that DON significantly increased IL-1β levels when both experimental regimens were considered together (*p* = 0.04), with no interaction between regimen and treatment (*p* = 0.09). All results are presented in Table 5.

**Table 5:** Inflammatory profile in the testes of prepubertal and adult mice exposed to a control or DON-contaminated diet for 15 days and 28 days, respectively

Analysis	Prepubertal Control	Prepubertal DON	Adult Control	Adult DON	Mean Control	Mean DON	Treatment effect *p*-value	Regimen effect *p*-value	Treatment × regimen *p*-value
NAG activity (number of macrophages/mg tissue)	658,136.10 ± 37,130.99	444,140.86 ± 49,700.00	473,964.50 ± 66,554.89	898,151.15 ± 310,037.84*	566,050.30	671,146.00	0.19	0.22	0.017
IL-1β (pg/mg protein)	28.24 ± 4.14	44.21 ± 16.71	8.26 ± 5.84	9.66 ± 4.01	18,20	24,90*	0.04	< 0.0001	0.09
TNF-α (pg/mg protein)	31.85 ± 8.59	31.58 ± 12.55	17.71 ± 3.23	39.53 ± 9.76*	24,80	35,60	0.05	0.57	0.04

Values are expressed as mean ± standard deviation. “Mean Control” and “Mean DON” represent the respective means across the prepubertal and adult experimental regimens. *p*-values for the effects of treatment, experimental regimen, and the treatment-by-regimen interaction were obtained using two-way ANOVA (*p < 0.05);. When a significant interaction was detected, the control and DON groups were compared within each experimental regimen using Šídák’s multiple-comparisons test (*p = 0.03 for the NAG activity and *p = 0.02 for the NAG activity and TBARS analysis, respectively)

### Daily sperm and sperm morphology

Many factors affect either DSP or morphology, including toxins, infections, and genetic conditions, which can lead to reduced sperm count and abnormal sperm shapes (Yang et al. 2019). The DSP and sperm morphology of adult animals were evaluated after DON exposure, revealing a significant reduction of 20% in DSP (*p* = 0.0049) and a significant increase of 31.37% in the rate of abnormal sperm (*p* < 0.0001) (Table 6).

**Table 6:** Sperm production and morphology of adult mice exposed to a diet contaminated with 10 mg of deoxynivalenol (DON) for 28 days

	Control	DON	*P*-value
Daily sperm production (x10^6^ / g testis / day)	46.48 ± 3.05	36.22 ± 2.23 **	0.0049
Abnormal sperm (%)	1.02 ± 0.03	1.34 ± 0.03***	< 0.0001

Values expressed as mean ± S.E.M. Student’s t-test. ** *p* < 0.01; *** *p* < 0.0001

## Discussion

Human and animal exposure to DON is a global concern due to its widespread occurrence and significant associated economic impact (Awuchi et al. 2021). In this context, our research group recently published a review article that compiled and synthesized the available evidence on the toxic effects of DON on male reproduction (Gomes et al. 2025), highlighting alterations in steroidogenesis (Yang et al. 2024), impairment of the BTB (Miao et al. 2023), and impairments in sperm parameters (Cao et al. 2020). However, this review revealed a significant gap in the literature regarding the effects of DON on young males, especially during the prepubertal phase, with only two studies available (Gerez et al. 2021; Yang et al. 2024). In this context, the present study provides evidence of morphological, morphometric, oxidative, and inflammatory alterations in the testes of prepubertal and adult mice exposed to DON, as well as impairments in sperm quantity and quality observed in adult animals.

It should be noted that an important limitation of the present study is the difference in exposure duration between prepubertal and adult mice, which are difficult to disentangle age-related effects from differences in exposure duration. In prepubertal mice, the 15-day exposure period was selected to encompass the peripubertal developmental window (Picut et al. 2018). In contrast, adult mice were exposed for 28 days, consistent with the conventional duration used in repeated-dose toxicological studies in adult rodents (OECD 2008). Another relevant limitation is the inability to determine individual DON intake across the different developmental stages, as the animals received the contaminated feed *ad libitum* and individual feed consumption was not recorded. Consequently, the actual amount of DON ingested by each animal could not be determined.

In this study, prepubertal mice exposed to DON exhibited a lower final body weight compared with their control. This finding is consistent with previous studies conducted in prepubertal female mice and prepubertal male rats exposed to the same DON concentration in the diet used in the present study (10 mg DON / Kg of feed), although the exposure periods differed between studies. In female mice, exposure lasted 15 days (Kono et al. 2026), whereas male rats were exposed for 28 days (Gerez et al. 2022). These findings suggest that DON exposure may adversely affect body weight gain during early development. The reduction in body weight observed following DON exposure may be related to the well-established anorexigenic effects of this mycotoxin (Awuchi et al. 2021). In addition, DON-induced gastrointestinal disturbances may contribute to impaired body weight due to intestinal barrier disruption and gut microbiota dysbiosis (Kępka-Borkowska et al. 2025). Furthermore, DON is known to inhibit protein synthesis, a mechanism that may impair tissue growth and contribute to reduced weight gain during development .

The testes are composed of seminiferous tubules, which contain germ cells and Sertoli cells (İnanç et al. 2025). Measurements of tubular diameter and seminiferous epithelium height are useful indicators of cellular loss or proliferation within the tubules, as well as when counting Sertoli cells (Suleimen et al. 2025). In the present study, the diameter of the seminiferous tubules was significantly reduced in adult animals, with a significant interaction between treatment and experimental regimen. Furthermore, there was also a reduction in germinal epithelium height when both experimental regimens were considered together, with no interaction between regimen and treatment. These findings are consistent with previous reports showing a reduction in tubular diameter and epithelial height in six-week-old mice exposed to 2.8 mg/kg of DON by oral gavage for 28 days (Yang et al. 2023). Such alterations may explain the decreased testicular weight described in previous studies involving broiler breeder roosters receiving 12.6 mg/kg DON-contaminated feed for 12 weeks (Yegani et al. 2006), broiler chickens fed 5 mg/kg DON-contaminated feed for 35 days (Froment et al. 2017), mice exposed to 2.4 mg/kg body weight for 4 weeks by oral gavage (Yang et al. 2019), and mice exposed to 2.4 mg/kg DON for 28 days by oral gavage (Cao et al. 2020).

Additionally, no significant changes were observed in the number of Sertoli cells. Given that the seminiferous epithelium comprises both Sertoli and germ cells, the reduction in seminiferous epithelial height observed in the present study, together with the preservation of Sertoli cell numbers, is consistent with a reduction in the germ cell population. This alteration may contribute to the decreased sperm counts observed in adult animals. However, because germ cell populations were not quantified separately, this interpretation should be considered a possible explanation rather than a direct demonstration of germ cell loss. The absence of changes in Sertoli cell numbers of contrasts with the results of Gerez et al. (2021), which reported a reduction in these cells in prepubertal rats exposed to similar doses of DON (9.4 mg/kg DON) for 28 days through *ad libitum* feed administration. The shorter exposure period in our study may explain this discrepancy.

To elucidate the mechanisms underlying germ cell loss, immunohistochemical analysis of caspase-3 was performed. These findings suggest that increased caspase-3 activation was not evident in the present experimental model; however, the evaluation of various apoptosis-related enzymes is necessary to precisely determine the involvement of apoptosis in DON-induced germ cell loss. Recent evidence indicates that DON can induce pyroptosis in the testes, a type of inflammatory cell death, contributing to germ cell damage. In this context, future studies exploring this pathogenic pathway are needed to elucidate the cellular mechanisms involved in the testicular effects of DON in prepubertal and adult individuals (Song and Zhang 2021).

Interestingly, despite the unaltered number of Sertoli cells, testicular morphology was affected in both age groups, suggesting a possible disruption of the BTB. In the morphological evaluation, the increased number of germ cells within the tubular lumen and the vacuolization of the germinal epithelium further supports the hypothesis of BTB impairment. DON has been shown to downregulate BTB junctional proteins in adult animals, such as mice exposed to 2.4 mg/kg by oral gavage for 28 days (Cao et al. 2020), and our findings suggest that a similar mechanism may also occur in prepubertal individuals. However, further studies evaluating the expression of BTB-associated proteins are warranted to confirm this hypothesis. Previous studies have demonstrated that DON increases testicular injury scores in adult animals, including mice exposed to 2.4 mg/kg for 28 days (Cao et al. 2020), mice treated with 12 mg/kg for 35 days (Li et al. 2023), rats exposed to 2.5 mg/kg for 28 days (Sprando et al. 2005), and mice treated with 2.0 mg/kg for 28 days (Yang et al. 2023). However, the present study is the first to evaluate these parameters in prepubertal mice.

It is important to note that, in this study, no significant changes were observed in the number of Leydig cells, which contrasts with the findings reported by Gerez et al. (2021) in prepubertal rats exposed to 9.4 mg/kg DON for 28 days through *ad libitum* feed administration. Differences in species and exposure duration may account for these discrepancies, as previous studies have shown that DON toxicity is highly species-dependent and dose- and time-dependent. For example, 56-day-old rats exposed to DON–contaminated feed containing 1.75 mg/kg for 30 days or 11.4 mg/kg for 7, 14, or 30 days – show variable histopathological and immunological responses (Bracarense et al. 2017). Moreover, variations in species-specific metabolism may result in distinct sensitivity levels to DON among animal models (Eriksen and Pettersson 2004).

The dynamics of spermatogenesis showed alterations when both experimental regimens were considered together, characterized by a reduction in the proportion of seminiferous tubules in stages VII–VIII. These stages correspond to important events in spermatogenesis, especially related to the final maturation of spermatids and spermiation. Thus, this alteration suggests a delay in the onset of spermiation (Carvalho et al. 2022) and it may compromise the normal progression of spermatogenesis, potentially resulting in a delay in the onset of puberty and morphological alterations in spermatozoa (O’Donnell et al. 2011). Furthermore, changes in the dynamics of the seminiferous epithelium cycle are frequently associated with hormonal disorders (Beardsley and O’Donnell 2003). In this context, although our study did not evaluate serum levels of reproductive hormones, previous studies have demonstrated that DON can reduce testosterone levels (Cao et al. 2020). Therefore, the alterations in spermatogenic dynamics observed in the present study are consistent with the possibility that endocrine changes may contribute to the effects of DON on spermatogenesis.

Testicular macrophages play a crucial role in maintaining the immunosuppressive environment of the testes through the production of anti-inflammatory cytokines, such as IL-10 and TGF-β (Loveland et al. 2017). Alterations in macrophage populations can disrupt testicular immune homeostasis, promote inflammation, and elevate levels of pro-inflammatory cytokines (Dutta et al. 2021). Notably, the pro-inflammatory cytokines are not exclusively expressed by immune cells such as testicular macrophages (Lysiak 2004); they are also produced by Leydig cells and germ cells and exert functions beyond immunological regulation, including roles in steroidogenesis and the restructuring of the BTB (Hedger and Meinhardt 2003). Consequently, alterations in cytokine levels may contribute not only to pro-inflammatory tissue status but also to modifications in hormone production and seminiferous epithelium integrity (Li et al. 2009).

In the present study, such alterations were evidenced in the testes of adult mice by an increase in the number of macrophages and higher levels of the cytokine TNF-α. Furthermore, it was observed that DON also significantly increased IL-1β levels when both experimental regimens were considered together. These changes could explain the higher proportion of morphologically altered seminiferous tubules, as well as the quantitative and qualitative changes in spermatozoa observed in the adults.

DON is well recognized for exerting its pathogenic effects, in part, through oxidative stress, with previous studies reporting testicular oxidative damage in mice exposed to 1.0 mg/kg DON for 28 days by oral gavage (Yang et al. 2023), as well as ovarian and uterine oxidative damage in prepubertal and adult mice exposed to 10 mg DON/kg contaminated feed *ad libitum* for 15 and 28 days (Lemos et al. 2024). Testicular oxidative stress in these studies was characterized by decreased antioxidant levels, increased free radicals and enhanced lipid peroxidation in mice exposed to 2.0 to 2.4 mg/kg body weight for 4 weeks by oral gavage (Yang et al. 2019, 2023). Our results demonstrate similar effects, with a reduction in antioxidant capacity (FRAP and ABTS) when the two regimens were considered together. Furthermore, superoxide anion levels (NBT) showed responses dependent on the experimental regimen, with prepubertal animals exhibiting higher levels of superoxide anion.

The findings of the present study, demonstrating testicular morphological and morphometric alterations associated with oxidative imbalance and a pro-inflammatory state, may explain the observed seminal changes in adult animals. Consistent with this interpretation, DON exposure has been shown to induce both quantitative and qualitative sperm alterations in mice following oral gavage administration of 2.4 mg/kg for 28 days (Cao et al. 2020).

These findings should be interpreted within the context of the established toxicological profile of DON and human exposure limits. The TDI for DON has been established as 1 µg/kg of body weight/day (Papageorgiou et al. 2018), whereas the dose employed in the present experimental model exceeds the maximum levels permitted by European regulations for infant foods—although such concentrations may occasionally occur in food products (Grenier and Applegate 2013).Importantly, dietary exposure assessments indicate that children may reach intake levels up to 3.5 times higher than the TDI, further highlighting the potential relevance of this mycotoxin in this population (Sundheim et al. 2017). Although the adverse effects of DON exposure are well established experimentally, this health risk in children and adolescents remains under-investigated. It is important to note that chronic exposure to low doses of DON has been shown to stimulate the immune system, inhibit protein synthesis, and suppress appetite, potentially contributing to impaired growth and developmental delays in children (Gonya et al. 2024). Collectively, these observations highlight the need for further studies examining the long-term consequences of early-life DON exposure, particularly with respect to reproductive development and function.

This study is among the few to comprehensively evaluate the effects of DON on male reproduction, encompassing both adult and prepubertal animals. The findings demonstrate that DON induces deleterious morphological and morphometric alterations in the testes at both experimental regimens. Notably, prepubertal animals exhibited greater susceptibility to oxidative stress, despite the shorter experimental duration and lower ingested dose of DON. However, the varying exposure durations between groups, the *ad libitum* administration of a single DON concentration, and the differences in intake between age groups limit the ability to distinguish effects related to developmental stage from those related to dose and experimental duration. Further studies employing multiple DON concentrations, as well as equivalent administration methods and exposure durations, are needed to clarify the dose-response relationship and distinguish developmental stage-dependent effects, thereby elucidating the consequences of DON exposure on male reproductive function.

## Data Availability

No datasets were generated or analysed during the current study.

## Declarations

### Artificial intelligence use

NotebookLM, an artificial intelligence tool, was used to create graphical abstract.
